# Insect-pathogen crosstalk and the cellular-molecular mechanisms of insect immunity: uncovering the underlying signaling pathways and immune regulatory function of non-coding RNAs

**DOI:** 10.3389/fimmu.2023.1169152

**Published:** 2023-08-24

**Authors:** Deepak Kumar Mahanta, Tanmaya Kumar Bhoi, J. Komal, Ipsita Samal, R. M. Nikhil, Amit Umesh Paschapur, Gaurav Singh, P. V. Dinesh Kumar, H. R. Desai, Mohammad Abbas Ahmad, P. P. Singh, Prasanta Kumar Majhi, U. Mukherjee, Pushpa Singh, Varun Saini, Shahanaz, N. Srinivasa, Yogesh Yele

**Affiliations:** ^1^ Department of Entomology, Dr. Rajendra Prasad Central Agricultural University, Samastipur, Bihar, India; ^2^ Forest Protection Division, Indian Council of Forestry Research and Education (ICFRE) - Arid Forest Research Institute (ICFRE-AFRI), Jodhpur, Rajasthan, India; ^3^ Department of Entomology, Navsari Agricultural University, Navsari, Gujarat, India; ^4^ ICAR-National Research Centre on Litchi, Mushahari, Ramna, Muzaffarpur, Bihar, India; ^5^ Division of Entomology, Indian Agricultural Research Institute, New Delhi, India; ^6^ Crop Protection Division, Indian Council of Agricultural Research (ICAR)-Vivekananda Parvatiya Krishi Anusandhan Sansthan, Almora, Uttarakhand, India; ^7^ The Directorate of Research, Maharana Pratap Horticultural University, Karnal, Haryana, India; ^8^ Department of Plant Pathology University of Agricultural Sciences, Bengaluru, Karnataka, India; ^9^ Department of Entomology, Main Cotton Research Station, Navsari Agricultural University, Gujarat, India; ^10^ Department of Entomology, Tirhut College of Agriculture, Dr. Rajendra Prasad Central Agricultural University, Samastipur, Bihar, India; ^11^ Department of Plant Breeding and Genetics, Odisha University of Agriculture and Technology, Bhubaneswar, Odisha, India; ^12^ Department of Entomology, Chaudhary Charan Singh Haryana Agricultural University, Hisar, Haryana, India; ^13^ Department of Entomology, College of Horticulture Mojerla, Sri Konda Laxman Telengana State Horticultural University, Wanaparthy, Telengana, India; ^14^ Department of Entomology and Agricultural Zoology, Institute of Agricultural Sciences, Banaras Hindu University, Varanasi, Uttar Pradesh, India; ^15^ School of Crop Health Management Research, Council of Agricultural Research-National Institute of Biotic Stress Management (ICAR)- National Institute of Biotic Stress Management, Raipur, India

**Keywords:** insect, insect-pathogen crosstalk, immune system, signaling pathway, effector mechanism, antimicrobial peptide

## Abstract

Multicellular organisms are constantly subjected to pathogens that might be harmful. Although insects lack an adaptive immune system, they possess highly effective anti-infective mechanisms. Bacterial phagocytosis and parasite encapsulation are some forms of cellular responses. Insects often defend themselves against infections through a humoral response. This phenomenon includes the secretion of antimicrobial peptides into the hemolymph. Specific receptors for detecting infection are required for the recognition of foreign pathogens such as the proteins that recognize glucans and peptidoglycans, together referred to as PGRPs and βGRPs. Activation of these receptors leads to the stimulation of signaling pathways which further activates the genes encoding for antimicrobial peptides. Some instances of such pathways are the JAK-STAT, Imd, and Toll. The host immune response that frequently accompanies infections has, however, been circumvented by diseases, which may have assisted insects evolve their own complicated immune systems. The role of ncRNAs in insect immunology has been discussed in several notable studies and reviews. This paper examines the most recent research on the immune regulatory function of ncRNAs during insect-pathogen crosstalk, including insect- and pathogen-encoded miRNAs and lncRNAs, and provides an overview of the important insect signaling pathways and effector mechanisms activated by diverse pathogen invaders.

## 1 Introduction

Insects are often attacked by pathogens (bacteria, fungi, viruses, etc.), and to defend themselves from these invaders, they have developed cellular and molecular defense systems against infection (1–5). Insects are equipped with physical barriers that prevent invaders from penetrating their hemocoel (1, 6). At the commencement of infections from bacteria, fungi, viruses, or protozoa, insect’s early defense mechanisms includes the production of antimicrobial compounds, identification of microbes by pattern-recognition receptors (PRRs), and the activation of diverse phagocytic cells which ultimately eliminates the invaders (7). These preliminary mechanisms are collectively referred to as “innate immune systems” (8). Insects’ innate immune responses are activated and coordinated by immunological components that have been remarkably conserved throughout evolution. This immunity comprises cellular and humoral responses (9). With particular receptors for microbial antigens, cells in vertebrates, including mammals, make it easier to identify microbes later on throughout the course of an infection (10). A variety of B lymphocytes and T lymphocytes are involved in adaptive immunity by recognizing infectious agents (11). Although insects lack an adaptive immune system, their innate immune system is still very effective at recognizing and targeting foreign substances (12).

The integument and peritrophic membrane constitute physical barriers. The cuticle forms the outermost covering of an insect’s integument, which consists of a single cell layer (13). Chitin and glycoprotein compose the peritrophic membrane, which covers the insect midgut (14). By creating a physical barrier, it safeguards the digestive system from harmful microorganisms and coarse food particles (14). Mucus, made up of glycosylated proteins, is another crucial physical structure (15). Also, the gut epithelial cells and acidic gastrointestinal tract both serve as natural barriers against microorganisms (16). Additionally, the differentiation of intestinal stem cells continuously promotes the repair of the natural barrier (17). A sophisticated and potent physical defense mechanism is produced by the interaction of the gut physical structure and stem cell development (14). The cellular and humoral immune responses are triggered when pathogens breach these boundaries (18).

Antimicrobial peptides (AMPs) are produced as a part of humoral immune mechanism (19). According to studies, the well-studied insect signaling pathways are immune deficiency (Imd), Toll, and Janus kinase/signal transducers and activators of transcription (JAK/STAT). Effector molecules like AMPs that have a tendency to annihilate pathogens invading insects may be produced when these pathways are triggered (20). Nevertheless, it was noted that pathogens may come into contact with these signaling pathways, which might result in their replication and proliferation (21). In contrast, cellular responses rely on insect hemocytes, which are involved in processes such as nodulation, encapsulation, phagocytosis, apoptosis, and autophagy (22). The fluid found in the hemocoel, known as hemolymph, carries nutrients throughout the insect body and is filled with a variety of different kinds of mobile cells called hemocytes (23). Hemocytes come in a variety of forms, such as granulocytes, spherulocytes, plasmatocytes and oenocytoids (24). Furthermore, it is essential to note that not every insect species possess all these hemocyte types (25). A decrease in circulating plasmatocytes, which make up nearly 95% of all hemocytes in *Drosophila melanogaster* larvae, after an infection illustrates the significance of hemocytes (26). Additionally, adult *Drosophila* are more vulnerable to microbial infections after having their phagocytic hemocytes eliminated either genetically or mechanically (18).

Once the hemocoel is infected, cellular immune responses begin almost immediately, while humoral immune responses take many hours to begin (27). It is hypothesized that hemocytes obliterate majority of the invading microorganisms before the remaining few are finally destroyed by humoral responses (28). These defensive systems interact with one another in order to function. Hemocytes, for example, produce molecules that promote interactions with microbes (29). These molecules aid in leukocyte phagocytosis in a manner similar to that of opsonins (complement and antibodies) (30). The production of antimicrobial peptides by fat body (the insect liver) cells is also triggered by plasmatocytes following bacterial infection in *Drosophila* (31). Further, plasmatocytes help to protect adult flies from bacterial infections by lowering their sensitivity to pathogens including *Bacillus subtilis*, *Escherichia coli* and most notably *Staphylococcus aureus* (28). These results demonstrate definitively that an effective crosstalk exists between cellular and humoral immunity in insects.

Subsequently, after infections, pathogens developed strategies to avoid the host immune reaction, which may have enabled insects advance the sophistication of their immune response systems (1). During insect-pathogen interaction, a number of elements, including the gut microbiota of the host insect, noncoding RNAs (ncRNAs) and nutritional stress modulate the immune system (32). Noncoding RNAs (ncRNAs) (nonprotein-coding RNAs) are RNA molecules that are incapable of encoding proteins (33). Numerous studies and reviews provided insights on how ncRNAs effect insect immunity (34). Invading pathogens trigger a variety of signaling pathways and effector mechanisms in insects, and this article provides an overview of these processes. Although over years, the studies on insects’ immunity, and related signaling pathways were becoming dominant (**Supplementary File**), most of the studies were published in research and review papers, and journals like journal of biological chemistry and developmental and comparative immunology were the forerunners in the insect immunity related papers. Although in the recent era studies have been increased over years, yet, this review focusses on following aspects, that would further enlighten knowledge on immune signaling system of insects. There is also a discussion of current results about the regulation of the immune system through insect-pathogen communication by ncRNAs, particularly microRNAs and long ncRNAs.

## 2 Cellular mechanisms (hemocytes, phagocytosis, encapsulation, melanization and nodulation) of insect immunity

**Figure 1:**
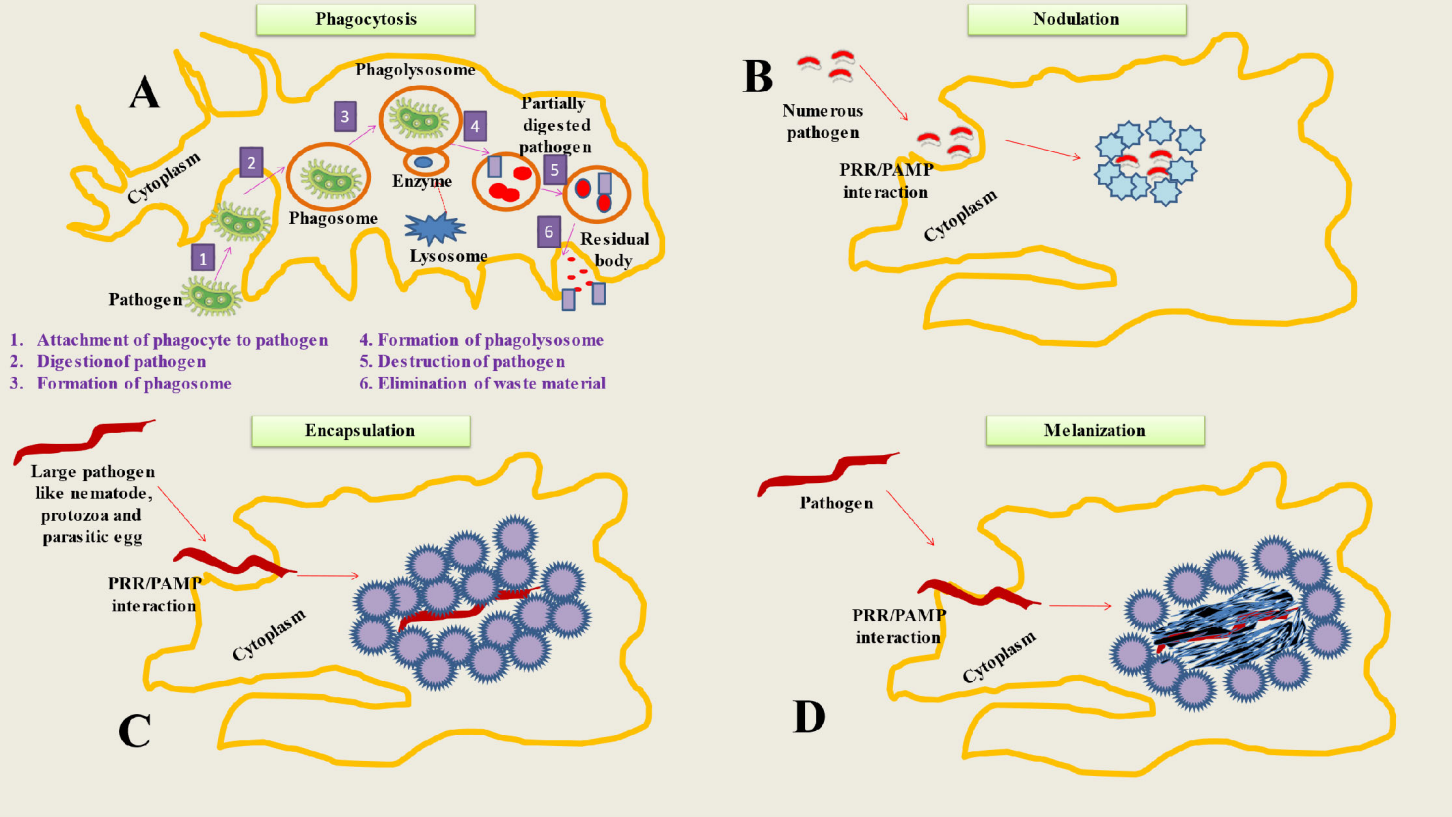
Represents Cellular mechanisms of insect immunity. **(A)** Phagocytosis is a process used by insects to neutralize and eliminate small pathogens. The phagocytes (hemocytes or granulocytes) mediate this process. The mechanism involve several steps like Attachment of phagocyte to pathogen, Digestion of pathogen, Formation of phagosome, Formation of phagolysosome, Destruction of pathogen and Elimination of waste material. **(B)** During the process of nodulation, granulocytes (immune cells) bind to one other to form layers that encase many bacteria or fungus spores. The bacteria are captured in a flocculent substance when the granulocytes discharge their contents. Melanization often happens after this stage. **(C)** When pathogens are too big to be phagocytosed, insects adopt encapsulation (both cellular and melanotic). Cellular encapsulation takes place without melanization, but melanotic humoral encapsulation depends on PO activity and may take place with or without the aid of hemocytes. **(D)** The process of melanization is based on the conversion of PPO to PO, that produces the melanotic capsule (melanotic enzymes) which facilitates the death of the foreign agent.

When the hemocoel is invaded, cellular immune responses happen right away, whereas humoral immune reactions take many hours to manifest (35). Hemocytes are in charge of a number of insect defensive mechanisms (36). Numerous variations in hemocyte immunological responses result due to the huge diversity of insect species (23). The majority of the examined insects do, however, exhibit a variety of regular cellular immune responses. Phagocytosis, encapsulation, nodulation, and melanization are some of these reactions (**Figure 1**) (37).

### 2.1 Hemocytes

There are different forms of hemocytes documented in insects, including prohemocytes, plasmatocytes, crystal cells, oenocytoids, and granular cells (38). These hemocytes possess adhesion and phagocytosis capabilities (39). While some forms of hemocytes such as oenocytoids, may produce prophenoloxidase (proPO) (40). The usual morphology-based categorization of hemocytes does not necessarily correspond with cell function (41). As a result, considerable effort has been put into classifying different hemocytes. Flow cytometry permits the grouping of three primary kinds of hemocytes: big granular cells, tiny semi granular cells and small hyaline cells (42). Additionally, some monoclonal antibodies can differentiate hemocytes based on their immunogenicity rather than their physical attributes (43). Also, these antibodies may also be able to inhibit other types of cellular responses (44). Three distinct hemocyte types in *D. melanogaster* have been characterized in further details: plasmatocytes, lamellocytes, and crystal cells (45). The large cells, known as crystal cells, earn their moniker from the crystalline inclusions they contain. During melanization, they release proPO, a zymogen that is essential for various physiological processes, including the closure of parasite eggs and the repair of damaged skin (22). About 95% of the hemocyte pool is comprised of plasmatocytes. They have relatively tiny (10 µm in diameter) cells, yet they produce enormous lamellipodial protrusions and active filopodia (46). Plasmatocytes are the persistent cells, that appear to remain throughout a fly’s life (47). Mature plasmatocytes were reported to express scavenger receptor ortholog Croquemort (Crq), phagocytic receptors, and the extracellular Peroxidasin (48). The lamellocytes were reported to only show up in the parasitized larval stages. One of the principal purposes of a hemocyte is to encase the parasitic wasp egg (49). With infection or sterile damage to wasp eggs, lamellocytes appear to develop from a pool of plasmatocytes that served as their predecessors (50). The first stage of the immune response in insects is the adhesion of granular hemocytes and plasmatocytes to the surface of the invading organism or to other cells (51). Phagocytosis, nodule formation, and encapsulation all result from hemocyte adhesion. Further the function of these innate cellular mechanisms are also explained.

### 2.2 Phagocytosis

Macrophages are a type of white blood cell that play a crucial role in the immune system, as they engulf and digest harmful particles and cells in the body (52). Recent advances in research have shed light on the evolution of macrophages, particularly the transition from invertebrate to vertebrate organisms (53). It is now believed that macrophages evolved from primitive, sessile cells found in invertebrates, which were then modified into motile cells in vertebrates (54). Additionally, studies have shown that different types of macrophages have evolved in different organisms, and that they play different roles in immune function (55). Understanding the evolution and function of macrophages can help improve our understanding of the immune system and the development of therapies for diseases (56). The principal role of macrophages entails the process of phagocytosis, which involves the engulfment and subsequent digestion of extraneous particles, including bacteria, viruses, and cellular remnants. This mechanism facilitates the eradication of pathogens by macrophages and their involvement in tissue repair processes. Moreover, macrophages are responsible for the synthesis and secretion of signaling molecules known as cytokines, which play a crucial role in the regulation of immune responses and facilitate communication among various immune cells (57). Gaining a comprehensive understanding of the complex functions of macrophages and their interactions with other immune cells yields significant insights into the pathogenesis and advancement of diverse pathological conditions. The dysregulation of macrophage activity has been linked to a variety of conditions, such as infectious diseases, autoimmune disorders, cancer, and chronic inflammatory diseases (58). Through the analysis of the molecular mechanisms that govern macrophage function, researchers have the opportunity to devise precise therapeutic interventions aimed at regulating macrophage activity. This approach holds promise for the treatment and prevention of various diseases. The phagocytosis of melanized bacteria and other small pathogens is common (**Figure 1**) (59). Phagocytosis is a cellular immunological mechanism that has been used by both vertebrate and invertebrate species throughout evolutionary history to prevent the spread of disease-causing microorganisms (60). It may phagocytose hundreds of bacteria at once and hydrolyze foreign bodies in a matter of seconds (61). Phagocytes including plasmatocytes and granulocytes, which can be either circulating or sessile are responsible for identifying foreign matter in Hemiptera, mosquitoes, Lepidoptera (granulocytes) and fruit flies (plasmatocytes) (62). The latter is taken up by a membrane-bound phagosome, which further fuses with a lysosome before being destroyed by enzymatic hydrolysis (46). The intracellular mechanisms driving phagocytosis are poorly understood, but it all starts with the binding of a cell-surface and humoral PRR on a PAMP (63). Nimrod proteins, Thioester-containing proteins, β-integrins, DSCAM and PGRPs are PRRs that have been experimentally shown to be associated with phagocytosis (64). There are several specifics among PRRs. For instance, NimC1 mediates the phagocytosis of *S. aureus* and, to a lesser degree, *E. coli*, whereas in *D. melanogaster* PGRP-LC mediates the phagocytosis of *E. coli* but not *S. aureus* (65).

### 2.3 Encapsulation

Encapsulation, a cellular immunological response, is deployed by insects to combat infections that are too huge to be phagocytosed (**Figure 1**) (66). Insects were found to exhibit two forms of encapsulation: melanotic humoral encapsulation (Diptera) and cellular encapsulation (Lepidoptera) (67). The latter may take place even in the absence of melanization (68). Contrarily, phenoloxidase (PO) activity is necessary for melanotic encapsulation, which may take place with or without hemocyte support (40). Granulocytes and plasmatocytes play a key role in encapsulation in Lepidoptera, whereas plasmatocytes and lamellocytes do so in *Drosophila* (69). In Lepidoptera, encapsulated substances were enclosed by both an external layer of plasmatocytes and an internal layer of granulocytes (36). Insects, such as lepidopteran and dipteran larvae, often utilize this response when infected with parasitoid wasp eggs (70). Encapsulation in Lepidoptera begins with the integrin dependent attachments of granulocytes to certain sites designated by an Arg-Gly-Asp (RGD) sequence (31). The granulocyte cells surrounding the pathogen are covered by several layers of plasmatocytes, and these plasmatocytes are further enclosed by an adhesive layer of more granulocytes (71). In contrast, plasmatocytes and lamellocytes are the cells engaged in a similar process in *Drosophila* (72). The capsule may subsequently get melanized depending on the infection and insect.

### 2.4 Melanization and nodulation

The insect immune system relies in part on melanization, an enzymatic process that serves a number of purposes (**Figure 1**) (73). To achieve this, serine proteases, its inhibitors, pattern recognition receptors, and enzymes involved in melanin synthesis should act together (36). In response to recognition of PAMPs by PRRs (C-type lectins, β-1,3 glucan recognition proteins and Gram-negative binding proteins), the serine protease cascade is activated, resulting in the conversion of pro-phenoloxidase (PPO) to phenoloxidase (PO), which in turn leads to melanotic capsule formation (74, 75). The pathogen’s proteinaceous capsule, in conjunction with damage, oxidative stress, or starvation, serves as a mediator in its annihilation (1). Additionally, melanization aids in the removal of infections (76). Oenocytoids, which are the main producers of PPO, are one kind of hemocytes that produce a number of enzymes and PRRs that trigger the process of melanization (1). Another crucial component of the insect’s immune system’s resistance against fungal infection is through melanization (77). Melanin, a dark pigment, is deposited at the site of infection. Upon detecting fungal pathogens, insects initiate their immune response, resulting in the release of specialized immune cells known as hemocytes. Hemocytes identify and phagocytose the invading fungi, initiating subsequent biochemical reactions (78). One reaction involves the activation of phenoloxidase, an enzyme that converts phenolic compounds to quinones. Quinones are subsequently polymerized and oxidized to generate melanin. Melanin deposition restricts the dissemination of fungal pathogens and hinders their capacity to induce harm (79). Moreover, melanin produces harmful by-products that negatively affect fungal cells. It is essential for containing and delaying the development and spread of the invasive mosquito pathogen *Beauveria bassiana* (80).

Although, the detailed molecular mechanisms defining this defensive process are still incompletely understood, nodulation depends on eicosanoid-based communication and the protein Noduler, which resembles an extracellular matrix (81). The first step in this procedure is the adhesion of granulocytes to one another to form layers that enclose many bacterial or fungal spores (1). When the granulocytes discharge their contents, a flocculent substance is created that traps the microorganisms. The nodule’s surface is then covered with an accumulation of plasmatocytes. Melanization often occurs after this stage (82).

## 3 The inducible humoral response of insect immunity upon pathogen invasions

The synthesis of antimicrobial peptides (AMPs) by insects is one of their earliest known defensive strategies (83). In response to microbial infection, the body secretes a combination of short peptides and proteins into the hemolymph (84). From almost undetectable in organisms to micromolar concentrations, AMP levels in the hemolymph of infected organisms rise sharply (85). Although hemocytes also contribute to the production of these AMPs, fat-body cells are the primary source of their expressions (86). Lysozyme, discovered in *Galleria mellonella*, was the first insect antimicrobial protein to be characterized (87). This enzyme found in the cell walls of Gram-positive bacteria is similar to C-type lysozyme of chicken that can hydrolyze peptidoglycans (88).

### 3.1 Antimicrobial peptides of insect immunity

As a result of biochemical analysis of the hemolymph of the fruit fly *Drosophila melanogaster* and other Dipterans, seven different types of AMPs have been revealed in insects (89). Based on their primary biological targets, they may be divided into three categories and exhibit a broad range of effects against microbes (90). Drosocin, attacins, cecropins and diptericin may all be used to combat Gram-negative bacteria. While, metchnikowin and drosomycin are antifungal medications (91). A characteristic feature of insect defensins is the presence of three or four stabilizing intramolecular disulfide bonds (92). The term derives from their chemical resemblance to mammalian α and β defensins (93). Insect defensins fall into two categories: those that include peptides with mixed α/β-helix-sheet structures and those that have triple-stranded antiparallel β-sheets (94). It has been revealed that numerous Lepidopteran species contain defensins with antibacterial and antifungal properties (95). Likewise, cecropins are short, basic peptides having an amphipathic α-helix shape, ranging in size from 31 to 37 amino acids (96). Cecropin is the first insect-derived amphipathic antimicrobial peptide discovered in the hemolymph of the silkworm *Hyalophora cecropia* (93). Cecropin was also observed to decrease proline absorption and cause membrane permeability by disrupting pathogen cell membranes and breaking the amphipathic peptides (97). Numerous Lepidopteran species have cecropin-family genes that have been identified. 13 cecropin genes have been discovered in *Bombyx mori* (98). Another category of amphipathic -helical antimicrobial peptides, moricins were initially identified in the silkworm, *B. mori* (99). Furthermore, nine moricin genes were found in the *B. mori* genome, while eight moricin homologs with antibacterial, antiyeast, and antifilamentous fungal activity were found in the *G. mellonella* genome (100, 101). Different terms, such as bactericidin, lepidopterin, and sarcotoxin, have been given to cecropins derived from insects besides *H. cecropia* (102). *D. melanogaster* produces the antimicrobial peptide drosocin, which is 19 amino acids long (93). The peptide’s antibacterial action has been traced to an O-glycosylated threonine residue since its absence significantly reduces the peptide’s potency compared to the original molecule (96).

The first known source of the 20 kDa AMPs called attacins, which are glycine-rich, was the hemolymph of *H. cecropia*. The acidic and basic isoforms of attacin have been cloned from *H. cecropia.* By primarily binding to lipo-polysaccharide (LPS), these attacins increase the permeability of the bacterial outer membrane (103, 104) and these AMPs were also reported to inhibit the synthesis of outer-membrane proteins by preventing the bacterial transcription (105). As a result, basic attacin was found to be more effective against *E. coli* than acidic attacin. *Spodoptera exigua*, the beet armyworm, is one of the several species of Lepidoptera whose attacins have been cloned (106). The Lepidopterans also have glycine-rich AMPs known as globerins and lebocins (107). These peptides prevent outer membrane protein production, which is essential for bacterial growth (108). The effectiveness of glomerins against bacteria, fungi, and even viruses has been demonstrated, and it has been hypothesized that they may also be able to inhibit viral replication (31).

Diptericin is a glycine-rich AMP that insects produce in response to bacterial infection or injury (109). It is basically a heat-stable peptide with an 8.6 kDa molecular weight with high concentrations of Asx, Pro and Gly (93). Although this treatment appears to work by rupturing the cytoplasmic membrane of growing bacteria, only a small subset of Gram-negative bacteria are susceptible to it (18). In addition to inhibiting bacterial growth, studies claim that diptericin also prevents oxidative stress (110). Through an increase in antioxidant enzyme activity in *D. melanogaster*, diptericin may “scavenge” or trap free radical anions as well as reduce oxygen toxicity (111). Drosomycin is a 44-residue antifungal peptide that was originally isolated from *D. melanogaster* exposed to microorganisms (112). It has been observed to be secreted from insect’s fat body into the hemolymph. It is quite effective against fungi but has little effect on bacteria. Drosomycin is a member of the cysteine-stabilized α‐helical and β‐(CSαβ) superfamily. It consists of a three-stranded α‐helix and a β‐sheet stabilized by four disulfide bridges (113). Moreover, it is highly similar to a class of 5 kDa cysteine-rich plant antifungal peptides identified in the seeds of diverse Brassicaceae species (114). Drosomycin has a narrow antibacterial range and is effective against just a few numbers of filamentous fungi (115). In *E. coli*, recombinant drosomycin was found to have antiyeast and antiparasitic activities (93). *Drosophila* produces the proline-rich peptide metchnikowin (26 residues) in response to infection (116). This peptide is produced in the adipose tissue in response to an immune challenge and its production may be initiated by the Toll or Imd pathways (117). Metchnikowin, a *Drosophila* based antimicrobial peptide (116) was recently demonstrated to have higher potential against Gram-positive bacteria and fungi. Further, transgenic barley containing the metchnikowin gene was successful in suppressing Fusarium head blight and powdery mildew like diverse ascomycetes fungal infections (118).

### 3.2 Signaling pathways activating antimicrobial peptide-encoding genes

**Figure 2:**
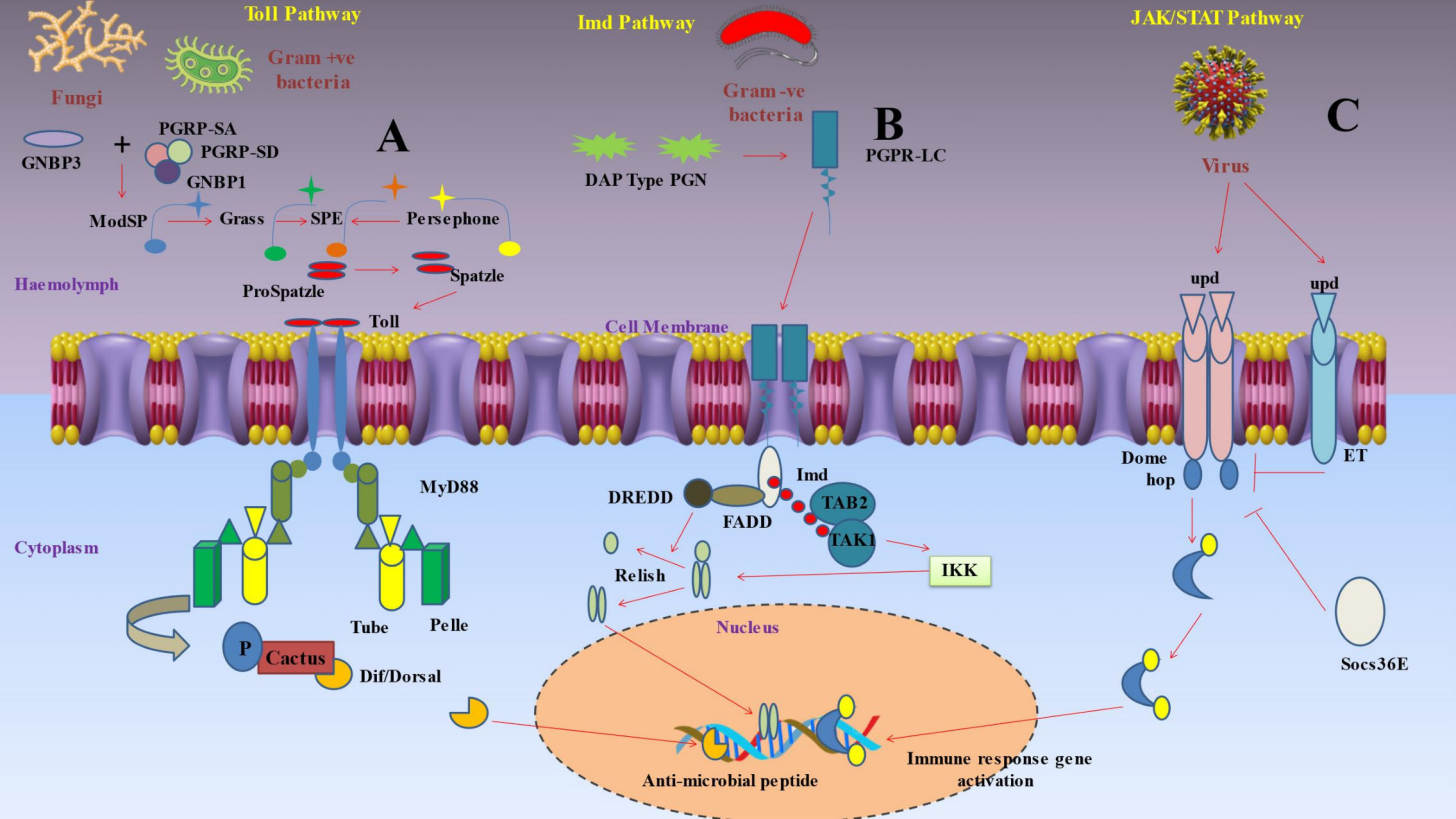
Depicts inducible humoral response of insect immunity upon pathogen invasions. **(A)** Protease cascades are crucial for activating Toll. When serine protease cascades cleave proSpätzle, Spätzle is produced as the Toll ligand. The circulating pathogen recognition receptor Gram-negative binding protein 3 (GNBP3) recognises the β‐1,3-glucan component of the cell walls of fungi, while the receptors peptidoglycan recognition proteins PGRP-SA and PGRP-SD, together with GNBP1, identify the peptidoglycan of Gram-positive bacteria. These interactions start protease cascades that culminate at the level of the serine protease ModSP, which subsequently triggers the protease Grass, that in turn activates the Spätzle digesting enzyme (SPE). The protease Persephone may also identify certain microbial proteases (virulence factors) produced by harmful fungus or bacteria. Persephone’s cleavage results in SPE activation and the development of active Spätzle. It takes a proteolytically cleaved version of Spätzle to activate the transmembrane receptor Toll. A signaling complex is put together when a dimer of Toll molecules recognises Spätzle. The TIR domains of Toll bind Myd88, while the death domains of Myd88 bind Tube and Pelle. In order to phosphorylate cactus (an IκB inhibitor), the kinase Pelle must first be activated by autophosphorylation. This marks the molecule for destruction. As soon as the NF-κB transcription factors Dorsal or Dif are liberated, they go to the nucleus and start the transcription of antimicrobial peptides (AMP). **(B)** Polymeric DAP-type peptidoglycan (poly PGN), which is identified by a dimer of PGRP-LC to activate Imd signaling, is found in several Gram-positive species as well as Gram-negative bacteria. The caspase DREDD (FADD death-related ced3/Nedd2 like protein) is then recruited when Imd attaches to FADD (Fas-associated protein with death domain). Imd is broken down by DREDD and activated by K63 ubiquitination. The TAK1-associated binding protein TAB2 and TAK1 (transforming growth factor beta (TGF‐β)-activated kinase 1) are both attracted to and activated by the K63 polyubiquitin chains. The IKK complex is then triggered by TAK1, which phosphorylates the nuclear factor Relish that is similar to NF-kB. In order to mediate the cleavage of the precursor relish, DREDD is also necessary. Free Relish may go into the nucleus after being broken down and phosphorylated, where it triggers the production of certain antimicrobial peptides (AMP). The intracellular receptor PGRP-LE may bind to monomeric peptidoglycan and trigger the Imd pathway. **(C)** Three cytokine-like proteins known as unpaired (upd), upd2, and upd3 transmit signals through the Domeless (Dome) receptor, that binds to a single JAK, hopscotch (hop). Following receptor activation, hopscotch phosphorylates itself as well as certain tyrosine residues on the cytoplasmic portion of the receptor. These phosphorylated tyrosines serve as docking sites for the Stat92E transcription factor, which is a component of the STAT family. Hopscotch phosphorylates Stat92E at tyrosine residues, enabling it to form dimers and translocate into the nucleus, where it binds the promoters of its target genes. This pathway is also under the control of a negative feedback loop involving the suppressor of cytokine signaling (SOCS) protein Socs36E, which is upregulated by STAT-JAK signaling. Moreover, eye transformer (ET), a nonsignaling receptor for upd, may interact with both Dome and hop as part of the receptor complex. Thus, it seems that ET inhibits intracellular signaling.

After pattern recognition receptors (PRRs) identify a microorganism, a series of signaling chemicals within the cells trigger various actions (**Figure 2**) (93). The eventual cellular response is determined by the molecule’s interactions with certain signaling pathways (119). Production of AMPs by the fat body through the Toll, immunological deficiency (Imd), and JAKSTAT pathways predominantly mediates humoral immune responses (120). The Toll signaling pathway is typically activated by gram-positive bacteria and fungi, while the Imd signaling system is generally triggered by gram-negative bacteria (121).

#### 3.2.1 The Toll signaling pathway

The Toll pathway was first discovered as a developmental pathway in *D. melanogaster* (122) and it included signaling to nuclear factor kappa B (NF‐κB), which is crucial for immunity and embryonic development (**Figure 2**) (123). The study of this pathway enables the characterization of toll-like receptors (TLRs), and by using this knowledge, it changed how we perceive the mammalian immune system (124). Insects depend on an intricate array of immune receptors for the detection and response to microbial infections. The Toll signaling pathway is a vital component of this network, as it plays a critical role in the defense against Gram-positive bacterial infections. The Toll pathway necessitates the involvement of additional pattern recognition receptors (PRRs). One example of a pattern recognition receptor (PRR) is Spatzle, an extracellular cytokine-like polypeptide that plays a crucial role in the activation of the transmembrane receptor Toll. Boraschi et al. (125) found evidence supporting the theory that Toll activation is dependent on the collaboration of other pattern recognition receptors (PRRs). Xiao et al. (126) discovered that a mutation in the peptidoglycan recognition protein (PGRP-SA), a specific pattern recognition receptor (PRR), inhibits the activation of the Toll signaling pathway by Gram-positive bacteria. This mutation significantly impairs the insect’s defense against Gram-positive bacterial infections. While PGRPs are responsible for mediating Toll activation, Gram-negative binding protein GNBP1 and GNBP3 were reported to be responsible for Gram-positive bacterial infections and fungal infections respectively (127). Subsequent studies on the Toll signaling pathway have provided further understanding of its mechanism of activation. Shang et al. (128) discovered that the protease Persephone, which is present in Drosophila, plays a vital role in the activation of the Toll pathway. Persephone undergoes proteolytic maturation through the action of fungal virulence proteins that are secreted. After reaching maturity, Persephone subsequently triggers the Toll receptor. This finding offers a novel understanding of the complex interaction between the host and invading fungi in the context of an infection. Additionally, Boraschi et al. (125) have highlighted that the cleaved version of the extracellular cytokine-like polypeptide Spatzle binds to the Toll receptor, thereby initiating Toll signaling. These findings provide insight into the complex activation mechanism of the Toll pathway, which includes protease maturation and the interaction between cleaved Spatzle and the Toll receptor. This interaction induces dimerization of intracytoplasmic TIR domains, which in turn induces binding of adaptor protein MyD88 via its own TIR domain (93). Pelle, a protein kinase, is employed when MyD88 links to the adaptor protein Tube (129). These interactions are established through direct contact between the death domains of the involved proteins. Autophosphorylation of Pelle upon binding causes it to promote the phosphorylation and degradation of cacti (an inhibitor of IB) and the nuclear translocation of the NFB transcription factors Dorsal and Dif (130).

#### 3.2.2 The Imd signaling pathway

Decreased AMP synthesis in response to *E. coli* and *Micrococcus luteus* infection led researchers to the *D. melanogaster* Imd (immunodeficiency) pathway (**Figure 2**) with this mutation alone (131). The antifungal Drosomycin, however, remained to be inducible in these flies. Subsequent studies demonstrated that the Toll pathway controlled Drosomycin induction after fungal infection, while the Imd mutation blocked the response to the majority of Gram-negative bacteria (132). The Imd pathway was reported to be activated by the binding of peptidoglycan recognition protein (PGRP)-LC and PGRP-LE to meso-diaminopimelic acid (DAP)-type peptidoglycan in the cell wall of most Gram-negative bacteria (133). These receptors trigger signaling to the NF-B transcription factor Relish via the death-related ced3/Nedd2 like protein (DREDD), Fas-associated protein with death domain (FADD), and transforming growth factor beta (TAK1), inhibitor of B kinase (IKK) signaling pathways (130). These receptors apparently dimerize after being coupled to peptidoglycan and connect to the adaptor protein Imd (93, 134). Imd activates the DREDD caspase and dFADD (*Drosophila* FADD) (135). IAP2 (Inhibitor of Apoptosis 2) is a ubiquitination system component that is activated when DREDD cleaves Imd, which is then K63ubiquitinated (129). K63polyubiquitin chains are thought to bind, recruit, and activate TAK1 via using the ubiquitin-binding domain of its regulatory companion TAK1-associated binding protein 2 (TAB2) (136). TAK1 then activates the IKK complex, allowing Relish to enter the nucleus. DREDD is also needed to help with the cleavage of the relish precursor (93).

#### 3.2.3 The JAK‐STAT signaling pathway

Similar pathways to Toll and Imd pathways were then discovered in mammals but the principal components of these pathways have stayed unchanged throughout evolution (137). The Janus kinase-signal transducer and activator of transcription (JAK-STAT) signaling pathway (**Figure 2**), on the other hand, was the first to be recognized as being pivotal in regulating a variety of aspects of human immunity, such as the control of inflammation and the activation of diverse leukocytes like neutrophils and macrophages (138). Although determining the *in vivo* roles of the JAK/STAT pathway and its regulators in mammalian systems has been difficult, scientists have conducted detailed studies by using fruit fly as the key model (93). The traditional model suggested that in JAK-STAT pathway, when the receptor binds to cytokine, it gets dimerized and activated (139). The activated JAKs further phosphorylate particular tyrosine residues that ultimately act as docking sites for the Src homology 2 (SH2) domains of STAT molecules (140). The JAK tyrosine phosphorylation of STATs was found to induce dimerization and nuclear translocation, and they finally get attached to the promoters of their respective target genes (141). This pathway is exceedingly intricate in humans due to the wide range of cytokines that can activate it, as well as JAKs and STATs’ ability to form homo- and heterodimers and bind with a number of transcription factors and coactivators (142). Four JAKs (JAK1, JAK2, JAK3, and TYK2) and seven STATs (STAT1, STAT2, STAT3, STAT4, STAT5A, STAT5B, and STAT6) were reported to be present (143). Only three cytokine like proteins termed unpaired (upd), upd2, and upd3 are recognized as JAK-STAT pathway ligands in *Drosophila* (144). A single JAK, hopscotch (hop), and a single STAT transcription factor, Stat92E, are bound by a single receptor, domeless (Dome), and are employed to signal by all three upd molecules (145). Additionally, the membrane-spanning signal transducer protein gp130, as well as negative feedback loops involving suppressor of cytokine signaling (SOCS) proteins, regulate the JAK-STAT pathway at the receptor level in mammals (93). Similar regulatory mechanisms have been discovered in *Drosophila*. The receptor complex contains the eye transformer (ET), a no signaling protein that mimics gp130 and interacts with both Dome and hop (146). Consequently, ET seems to prevent intracellular signaling (147). Additionally, the SOCS family has three members in *Drosophila*: Socs16D, Socs36E, and Socs44A. Among these, Socs36E is the main regulator of the negative feedback loop and is substantially triggered by JAK/STAT signaling (93). In *Drosophila*, the Toll and Imd pathways play a major role in regulating the humoral immune response, which results in the synthesis of antimicrobial peptides (110). Additionally, the JAK/STAT pathway triggers the fat body to produce other proteins, such as stress response proteins and cytokines. The ligand upd3 triggers the activation of this pathway. Hemocytes are induced to release upd3 under a variety of stress situations, including injury, heat shock, and dehydration (148). Additionally, it has been demonstrated that the JAK/STAT pathway plays a role in *Drosophila* viral response. Many viruses, including TotM, upd2, and upd3, boost the expression of well-known JAK/STAT pathway target genes (149). Finally, the JAK/STAT pathway stimulates the production of antimicrobial peptides in the stomach, including drosomycin-like peptide (dro3) (150). Rather than the pathogen, the recognition of cell injury appears to be the mediating mechanism in this response.

## 4 Receptors sensing infections

**Figure 3:**
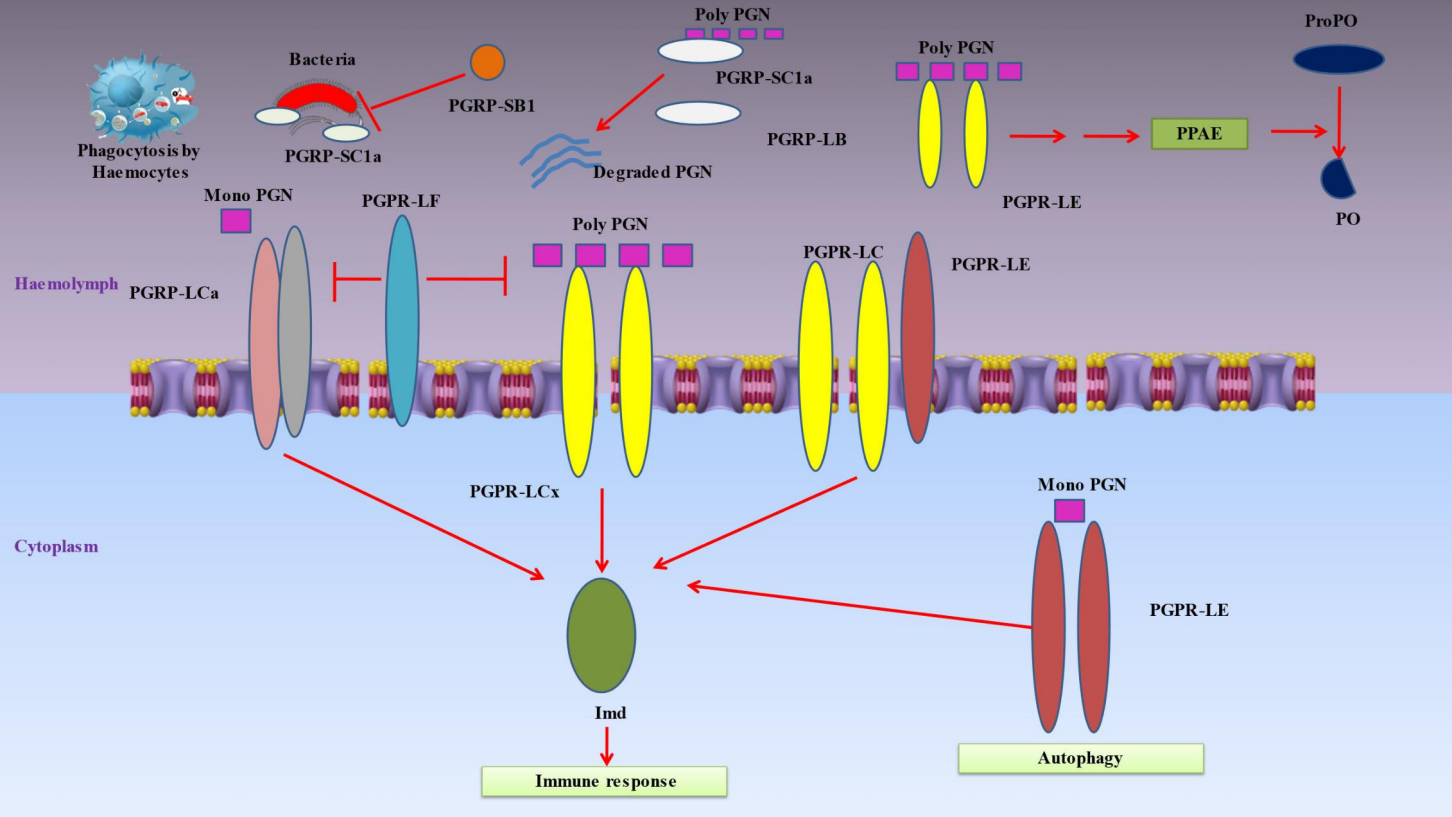
Denotes immune responses in insects when hemocyte receptors or plasma proteins attach to certain chemicals found on the surface of many different types of bacteria. Pattern-recognition proteins may be divided into a number of categories, such as peptidoglycan recognition protein (PGRP), β‐1,3-glucan recognition protein (β-GRP), C-type lectins and hemolin. Peptidoglycan recognition proteins (PGRPs), conserved from insects to humans, are innate immunity proteins that can identify bacterial peptidoglycan. When the PGRP-LCx homodimer complex contacts polymeric peptidoglycan (poly PGN) or the PGRP-LCx/PGRP-LCa heterodimer binds monomeric peptidoglycan, the Imd pathway is triggered. PGRP-LE is capable of binding polymeric and monomeric peptidoglycan. The prophenoloxidase (proPO) cascade is activated by extracellular PGRP-LE, which is also implicated in activating the Imd pathway via PGRP-LC transmembrane receptors and proPO cascade upstream of the proPO activating enzyme (PPAE). By identifying intracellular monomeric peptidoglycan and activating Imd signaling or autophagy without the help of Imd, intracellular PGRP-LE may also activate the Imd pathway. The Imd pathway is inhibited by PGRP-LF. The DAP-type peptidoglycan is broken down by PGRP-LB and SC1a into inactive fragments, which prevents the Imd pathway from being activated. Furthermore, PGRP-SC1a functions as an opsonin for bacterial phagocytosis. Due to its particular amidase activity for DAP-type peptidoglycans, PGRP-SB1 is immediately bactericidal.

Insect’s innate immune responses are triggered when hemocyte receptors or plasma proteins attach to certain chemicals found on the surface of many different types of bacteria, such as lipids or sugars (18). Pattern-recognition proteins may be divided into a number of categories, such as peptidoglycan recognition protein (PGRP), β‐1,3-glucan recognition protein (βGRP), C-type lectins and hemolin (**Figure 3**) (151).

### 4.1 Peptidoglycan recognition proteins

Peptidoglycan recognition proteins (PGRPs), which play a key role in inflammation and antibacterial defense by recognizing bacterial peptidoglycan, are essential proteins associated with innate immunity (**Figure 3**) (152). They are polymorphonuclear leukocyte-expressed (PGRP1), liver-expressed (PGRP2) or secreted proteins (PGRP3 and PGRP4) (93). Up to 19 PGRPs exist in insects, categorized as short (S) and long (L) variants (153). The short versions are found in hemolymph, cuticle and fatbody cells, while the long forms are mostly expressed in hemocytes (154). Insect PGRP expression is frequently elevated when subjected to bacterial exposure. These receptors cause proteolytic cascades that result in the production of antimicrobial compounds, activate the Toll or Imd signal transduction pathways, or both (155). The following are PGRPs in *Drosophila* that have known uses: The hemolymph’s PGRP-SA binds to Lys-type peptidoglycan, and together with the PGRP-SD and GNBP-1, this causes the Toll pathway to be activated (28). In reaction to yeast, GNBP3 also causes the Toll pathway to be activated (156). These pattern recognition proteins trigger a series of serine protease cascades that ultimately activate the Spatzle-processing enzyme (SPE), which cleaves proSpatzle to create free Spatzle, the ligand for Toll (157). In a similar way, the Imd pathway is triggered by the binding of DAP-type polymeric peptidoglycan to the PGRP-LCx homodimer complex or DAP-type monomeric peptidoglycan to the PGRP-LCx/PGRP-LCa heterodimer (158). Both monomeric and polymeric DAP-type peptidoglycans are capable of being bound by PGRP-LE (159). It has been shown that extracellular PGRP-LE activates the Imd pathway through PGRPLC transmembrane receptors and is also important in activating the prophenoloxidase (proPO) cascade ahead of the proPO-activating enzyme (PPAE) (160). By binding to the Imd adaptor protein, PGRP-LE inside the cell is able to activate the Imd pathway in response to the recognition of DAP-type peptidoglycan from bacteria within the cell. Furthermore, PGRP-LE produced within the cell may stimulate autophagy in a way that is independent of the Imd signaling pathway (161). PGRP-LF inhibits the Imd pathway because it binds to PGRP-LCx instead of peptidoglycan. By doing so, it inhibits the development of an active dimer of PGRP-LC. DAP-type peptidoglycan is cleaved into inactive pieces by PGRPLB and SC1a/1b/2, which prevents Imd pathway activation (162). As a result of its specialized amidase activity on DAP-type peptidoglycans, PGRP-SB1 is very lethal to bacteria (163).

### 4.2 Beta‐1,3‐glucan recognition proteins

A family of plasma proteins known as insect β‐1,3-glucan recognition proteins (GRPs) and Gram-negative bacteria binding proteins (GNBPs) have glucan-binding domains at their amino and carboxyl ends that are comparable to those of β‐1,3-glucanases (164). All βGRPs may trigger the proPO cascade (**Figure 3**) by attaching to β-1,3-glucans on bacterial surfaces. *Manduca sexta* βGRP1 gene expression is constant in the fat body, but βGRP2 gene expression is elevated during the initial wandering stage just before pupation or after an immunological attack (165). These βGRPs bind to the hemolymph proteinase-14 precursor (proHP14), which causes HP14 to autoactivate and start a proteinase cascade that activates proPO (166). *Helicoverpa armigera* larval midgut extract was used to identify a βGRP with glucanase activity. This enzyme likely serves as a digestive enzyme rather than an immune stimulator since it hydrolyzes β-1,3-glucan but not β-1,4-glucan (167).

### 4.3 Hemolin and C‐type lectins

The plasma protein known as hemolin, which is frequently found in the adhesion molecules of vertebrates, contains four immunoglobulin (Ig) domains (168). Hemolin is present in many Lepidopteran species, especially *B. mori* (101), *Antheraea mylitta* (169), *Plutella xylostella* (170) and *Samia cynthia* (171), yet it hasn’t been seen in insects from other orders. Lipoteichoic acid and LPS from bacteria are bound by hemolin (172). Hemolin also binds to hemocytes, acting as a link between hemocytes and microorganisms and triggering phagocytosis or nodulation (37).

Animal C-type lectins (CTLs) are a vast class of molecules that recognize carbohydrates and bind ligands in a calcium-dependent way (173). Lepidopterans have been reported to include a number of C-type lectins, including immulectins 1, 2, 3 and 4 as well as LPS-binding protein (also known as CTL20), CTL10, CTL11, CTL19 and CTL21 (174). These lectins all contain two carbohydrate recognition domains, and based on their genes, it seems that Lepidoptera is the only insect group that has these particular sorts of lectins (175). The majority of Lepidopteran CTLs bind to lipoteichoic acid and bacterial LPS, causing bacterial and yeast agglutination. This is likely because each of the two carbohydrate-binding domains binds to sugar residues on the surface of nearby microbial cells (176). This microbial aggregation may help hemocytes fight off pathogens by phagocytosing them and forming nodules. By phagocytosing the pathogens and forming nodules, this microbial aggregation could aid hemocytes in their fight against them.

## 5 Antiviral insect immune response

Viruses may infect insects just like any other kind of organisms (12, 177). Some viruses only infect insect cells and are confined to them, whereas other viruses are spread to mammals by insects that bite them (8).Thus, understanding how insect’s innate immune systems protect themselves against viruses is of paramount importance from a medical and economic standpoint. The RNA interference (RNAi) pathway, which identifies double-stranded RNA (dsRNA) produced from viruses and produces small interfering RNAs (siRNAs), is the main mechanism of antiviral defense (178). These siRNAs in turn aim to degrade viral RNA, which prevents the virus from replicating (179). In addition, it has been shown that various innate antimicrobial pathways, including the Imd, Toll, and JAK-STAT pathways, are crucial for insect antiviral responses. For instance, it seems that the JAK-STAT pathway has a role analogous to that of the mammalian interferon system (180). Bystander cells that are not infected by the virus receive a signal from a virus-infected cell that activates this pathway, resulting in antiviral action. In summary, it has been demonstrated that a number of viral infections involve the autophagy process (181).

### 5.1 RNA interference pathway based immune response

The RNA interference (RNAi) pathway provides the most potent insect response to viral infection. Dicer2 (an endoribonuclease belonging to the RNase III family) and the protein R2D2 collaborate to detect double-stranded viral RNA (182). Later, Dicer2 snips the dsRNA into manageable duplex DNA pieces (of around 21 nucleotides) (183). The duplex is unwound, and a guide strand is chosen according to its complementarity with the other strands. The RNA-induced silencing complex (RISC), which has the RNase Argonaute as part of it, is subsequently loaded with the siRNA guide strand (184). Argonaut destroys target viral RNA by disintegrating the complementary guide strand. Several viruses produce RNAi suppressor proteins (1A proteins in Nodaviridae or B2 proteins in Dicistroviridae) that limit the action of the RISC during infection, thus indicating the significance of the RNAi pathway in the regulation of viral infections (185). The FHV B2 protein is a dimer that binds to dsRNA to inhibit Dicer2 from digesting it (186). DCV A1 protein functions similarly to FHV B2 by attaching to dsRNA (187); while Argonaute suppresses the RNAse activity of the 1A protein of the cricket paralysis virus (CrPV) by binding to it (188). When a virus lacks these proteins, the reproduction is inhibited thus enabling the insects to easily remove the infectious microbe propagule. Clearly, the flavivirus NS4B protein of Dengue virus 2 (DENV2) inhibits siRNA pathways in human and insect (Sf21) cells as well (93).

### 5.2 The autophagy immune pathway

Insects also use autophagy as an antiviral strategy; unlike Toll, Imd, and JAK-STAT pathways, this one does not rely on them (1). During autophagy, double-membrane vesicles called autophagosomes are formed inside of cells (189). Newly synthesized membranes, including fragmented organelles and protein clumps, are used to form these vesicles (190). Lysosomes then work along with the autophagosome to digest its cargo. Autophagy is activated in response to a wide variety of stress signals, including as food deprivation, infection, and the need for cellular repair (191). Consequently, autophagy, a form of cellular degradation, aids in nutrient recycling and keeps cells in balance (192). The autophagy signaling system includes the phosphoinositide 3-kinase (PI3K)-Akt pathway, which elevates levels of the autophagy inhibitor target of rapamycin (TOR) (193). Under normal development conditions, TOR is active and phosphorylates the Autophagy-Related (Atg) 13 protein several times. Reduced Atg1 kinase activity prevents Atg13 from interacting with Atg1, preventing this crucial autophagy regulator from doing its job (194). In the presence of hunger, Atg13 is rapidly dephosphorylated, allowing it to form a complex with Atg1 and activate it; this in turn reduces TOR activity. Atg1 then binds to more Atg proteins to form the PAS, which in turn initiates autophagy (195). Under normal development settings, different Atg proteins assemble at the PAS to generate cytoplasm to vacuole targeting (Cvt) vesicles, but under famine conditions, different Atg proteins assemble at the PAS to generate autophagosomes (196). When infected with vesicular stomatitis virus (VSV), *Drosophila* exhibit a reduction in the PI3K-Akt-TOR signaling pathway (197). Dueto the increased autophagy, viral replication is inhibited (198). The viral surface glycoprotein (VSV-G) was hypothesized to be the PAMP that induced this cellular response (199). Toll7, the *Drosophila* TLR ortholog, was found to detect VSV on the cell surface, according to recent studies (200). Toll7 signaling was triggered by VSV infection, and blocking this signaling led to elevated viral protein levels *in vitro* and pathogenicity *in vivo* (201).

### 5.3 Apoptosis

Apoptosis may be thought of as a sort of programmed cell death (202). A molecular complex is formed between the adaptor protein Ark and the caspase Dronc (203). Effector caspases like Drice and Dcp1 are activated by Dronc, and when they cleave proteins, they ultimately cause programmed cell death (1). Apoptosis has a role in the defense against baculoviruses in Lepidoptera (204). This process seems to be important in mosquitoe’s defense mechanisms against the West Nile Virus and the Sindbis virus (205), Additionally, phagocytosis of apoptotic cells was found to impart defense against the *Drosophila* C virus (129).

## 6 Role of ncRNAs in insect immunity during microbial invasions

**Figure 4:**
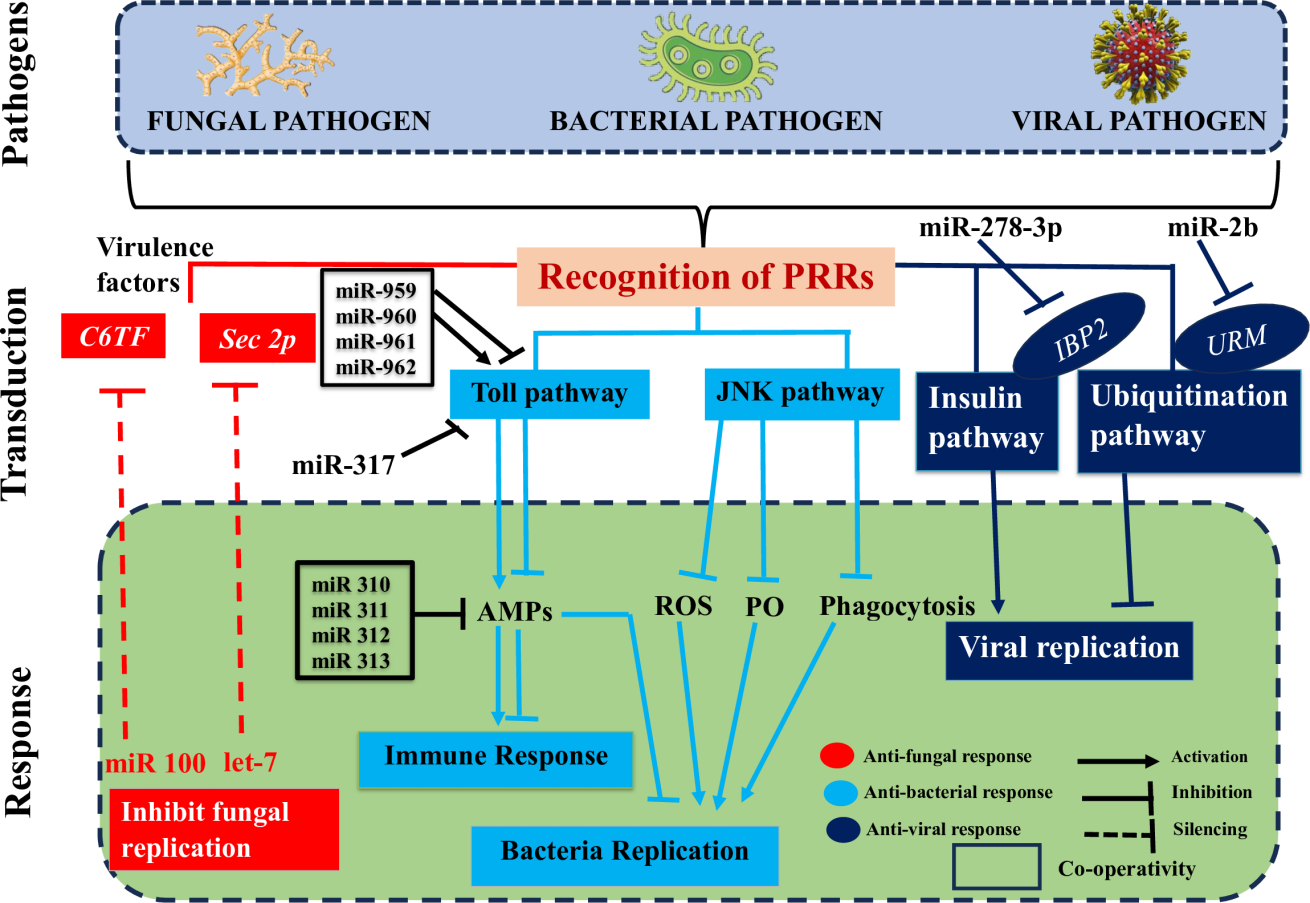
Deciphers how both insect and pathogen-encoded ncRNAs, particularly miRNAs play a role in immune regulation during insect-pathogen interaction. miRNAs alter the immunological response of insects to harmful pathogens. Insect miRNAs may silence fungal virulence genes, such C6TF and Sec2p, and prevent their replication in response to fungal invasion. Following bacterial invasion, insect miRNAs work alone or in concert to positively regulate the essential elements of the Toll pathway, which activates the AMP gene effectors and prevents bacterial replication. The expression of the AMP gene, which is necessary for insect immunological homeostasis, may be adversely modulated by those components in the late stage of infection. On the other hand, insect miRNAs (such as those found in the pea aphid *Acyrthosiphon pisum*) may block the JNK signaling pathway and indirectly those under its control (ROS, PO, and phagocytosis), which can boost bacterial multiplication. Finally, when viruses invade insects, IBP2 is markedly upregulated. In contrast, insect miRNAs prevent the replication of viruses by downregulating URMs, which are involved in the ubiquitination process.

Using high-throughput sequencing techniques and advanced bioinformatics tools, researchers have made significant progress in discovering and identifying novel insect ncRNAs and their controlled transcripts (206). There is substantial evidence that ncRNAs play a role in insect immunity, and that their expression levels are among the first to shift in response to microbial infections (**Figure 4**) (1). Here, we go beyond just discussing the differential up- or downregulation of these components in insects to emphasize the immunological targets of these changes and the impact they have on insect defenses against pathogen invasions. We will also discuss the role that pathogen-encoded ncRNAs play in modulating insect immunity. Insect-pathogen interaction has also been studied in terms of conserved and new microRNAs and long noncoding RNAs originating from insects and pathogens.

### 6.1 Role of miRNAs in insect immunity during microbial invasions

The interactions between a host and a pathogen are significantly influenced by miRNAs. Insects rely on the dynamic miRNA-mRNA for immunological response to pathogen attacks (**Figure 4**), as it is responsible for regulating the potent insect signaling pathways that either enhance or suppress innate immune responses ensuring homeostasis (1). For instance, an extensive variety of signaling pathways were enhanced after infection of *Drosophila* with *Micrococcus luteus* due to differentially produced miRNAs and mRNAs. These pathways included Toll and Imd (207).

#### 6.1.1 During fungal pathogen invasion

The insect-pathogen crosstalk is utilized to change the expression levels of miRNAs. It would be helpful to identify and characterize the miRNA’s cellular targets for a better understanding of the immunological modulatory function of these miRNAs. Insect immune systems can either combat infections by eliminating them from the host organism or neutralize them by preventing the production of toxins and virulence factors that promote invasion and destructive behavior of an intruder inside the host (**Figure 4**) (1). The virulence factors of invaders are suppressed by insect-encoded miRNAs, which seem to primarily deal with fungal diseases. This silencing disrupts several translation components connected to mRNAs with 5’-cap to 3’-tail structures (208). In a recent study, it was shown that when the fungus *B. bassiana* invades mosquito hemocoel, mosquitoes express higher amounts of let-7 and miR-100 miRNAs (209). The virulence-related genes sec2p and C6TF, which encode for a Rab guanine nucleotide exchange factor and a Zn (II) 2Cys6 transcription factor, respectively, are silenced by both miRNAs after they translocate into the fungal hyphae (209). According to theories, the insect miRNAs may be transported to the fungus-causing pathogen through extracellular vesicles (EVs).

#### 6.1.2 During bacterial pathogen invasion

Inhibiting immune signaling pathways is one way that miRNAs help restore immunological homeostasis in insects. Small interfering RNAs (**Figure 4**) (miRNAs), which inhibit the development of key components of the host’s immunological signaling pathways, play a role in this function individually or in concert. The tube is a critical effector molecule in the Toll pathway, together with the transcription factors Dl and Toll. During immune response of *Drosophila* to *M. luteus* infection, the miR-959-962 cluster acts synergistically with other miRNAs to target the 3’UTR of tube, dl, and Toll mRNAs, potentially inhibiting the synthesis of AMPs in the late stage of the infection. As a result, fewer flies would make it through the winter (210). Similar findings suggested that miR-960 may influence antibacterial defense only after a late 12-hour infection has set established. At 6 and 12 hours, miR-959 has the potential to persistently repress Dr expression. Yet, miR-961 may be more effective than miR-962 at dampening the immune system’s ability to fight off microorganisms. A potential miRNA, miR-958, was also identified via in silico screening when the Gal80ts-Gal4 driver system was used. This microRNA can regulate the signaling of the Toll pathway *in vitro* and *in vivo* by targeting the molecule Dif and the protein Toll in a negative fashion. There are four miR-958-binding sites in the Toll 3’UTR, and at site 3, miR-958 exhibits strong and specific inhibition (211). Toll signaling response in *Drosophila* was negatively regulated by miR-317, which acted in a manner similar to that shown here by targeting only the Dif-Rc isoform of the Dif four (212). Previous work by the same authors, however, showed that miR-317 controls the *Drosophila* Toll system by targeting the three extra Dif isoforms (Dif-Ra/b/d) (212). Several studies demonstrated that miR-317 plays a critical role in regulating reproductive responses and ovary development in *Drosophila* larvae (213, 214). Flies with transiently overexpressed miR-317 have a dismal chance of surviving. On the other hand, miR-317 knockout/wild-type (KO/+) flies fared better against Gram-positive bacterial infection than the control group did (212), suggesting a novel appreciation for miRNA’s part in the survival/immunity tango in *Drosophila*. In addition, four *Drosophila* miR-310 members (miR-310, miR-311, miR-312, and miR-313) directly cotargeted the 3’UTR of Drs and decreased the expression of Drs during Gram-positive bacterial infection, which had a negative effect on the Toll mediated immune response (211). In conclusion, insect-expressed miRNAs may act singly or in concert to dampen AMP expression and antibacterial defenses, thereby preserving immunological homeostasis (1). These methods not only facilitate the identification of hitherto unknown miRNAs, but also allow for the expansion of the insect repertoire of Toll-related immune-modulating miRNAs. They frequently involve regulating elements of the Toll pathway (1). Some hemipteran insect species, benefit from the conservation of genes creating immune effectors; however, other species, like the pea aphid *Acyrthosiphon pisum*, show diminished immunological responses due to a lack of these genes (215). It has been hypothesized that the pea aphid immune response employs the ubiquitous Jun N-terminal kinase (JNK) pathway (1). Ma et al. (216) looked at how this pathway regulates the pea aphid immune response to bacterial invasion and found that miRNA-184a/b suppressed the expression of JNK-3’UTR, which led to an increase in bacterial carriage and aphid mortality. After infection with *M. luteus* and *Pseudomonas aeruginosa*, miRNA-184a and miRNA-184b expression drastically dropped, reaching a minimum 24 hours after infection. This demonstrated a negative correlation between JNK expression and miRNA levels (1). Indirect evidence reveals that miRNA-184 controls the JNK pathway in the pea aphid, which is significant because it is regulated by PO, reactive oxygen species, and phagocytosis, all of which are involved in the aphid’s antibacterial immune response (1). Finally, the RNA hybrid programme predicted that JNK is a target of miRNA-184 in a wide variety of organisms, including insects, zebrafish, frogs, mice and humans. This finding suggests that miRNA-184 regulates the JNK pathway in a wide range of organisms (216). Those with UPEC strains in their bladders experience painful UTIs, while those with commensal-like *E. coli* strains in their bladders have chronic asymptomatic bacteriuria (ABU) (1). When studying human illnesses like UPEC, *G. mellonella* is used as a stand-in insect model host (217). As *G. mellonella* miRNA expression levels were considerably different the larvae challenged with UPEC strain CFT073 and ABU strain 83972, it is possible that miRNAs mediated by the insect immune response can distinguish between harmful and commensal *E. coli* invasions (1).

#### 6.1.3 During viral pathogen invasion

Host miRNAs play a crucial defensive role against viral attacks, which has an impact on the progression of the infection (**Figure 4**) (1). For instance, when *A. aegypti* is infected with the chikungunya virus (CHIKV), increased *A. aegypti* miR-2b binds to the 3’UTR of the ubiquitin related modifier (Urm), reducing translation. Finally, this causes *A. aegypti* to have lower CHIKV replication (218). On the other hand, there are also instances when insect miRNAs may encourage viral replication by suppressing the expression of virus-induced host genes (1). This is true for the insulin-related peptide-binding protein 2 (IBP2), which is well-known to be markedly increase in *B. mori* infected with viruses (219), but has been inhibited *in vitro* and *in vivo* by miR-278-3p, causing BmCPV replication. But the precise role of miR-278-3p and IPB2 in BmCPV replication has remained unclear, necessitating additional research in the future (220).

### 6.2 Role of lncRNAs in insect immunity after microbial invasions

#### 6.2.1 During fungal pathogen invasion

**Figure 5:**
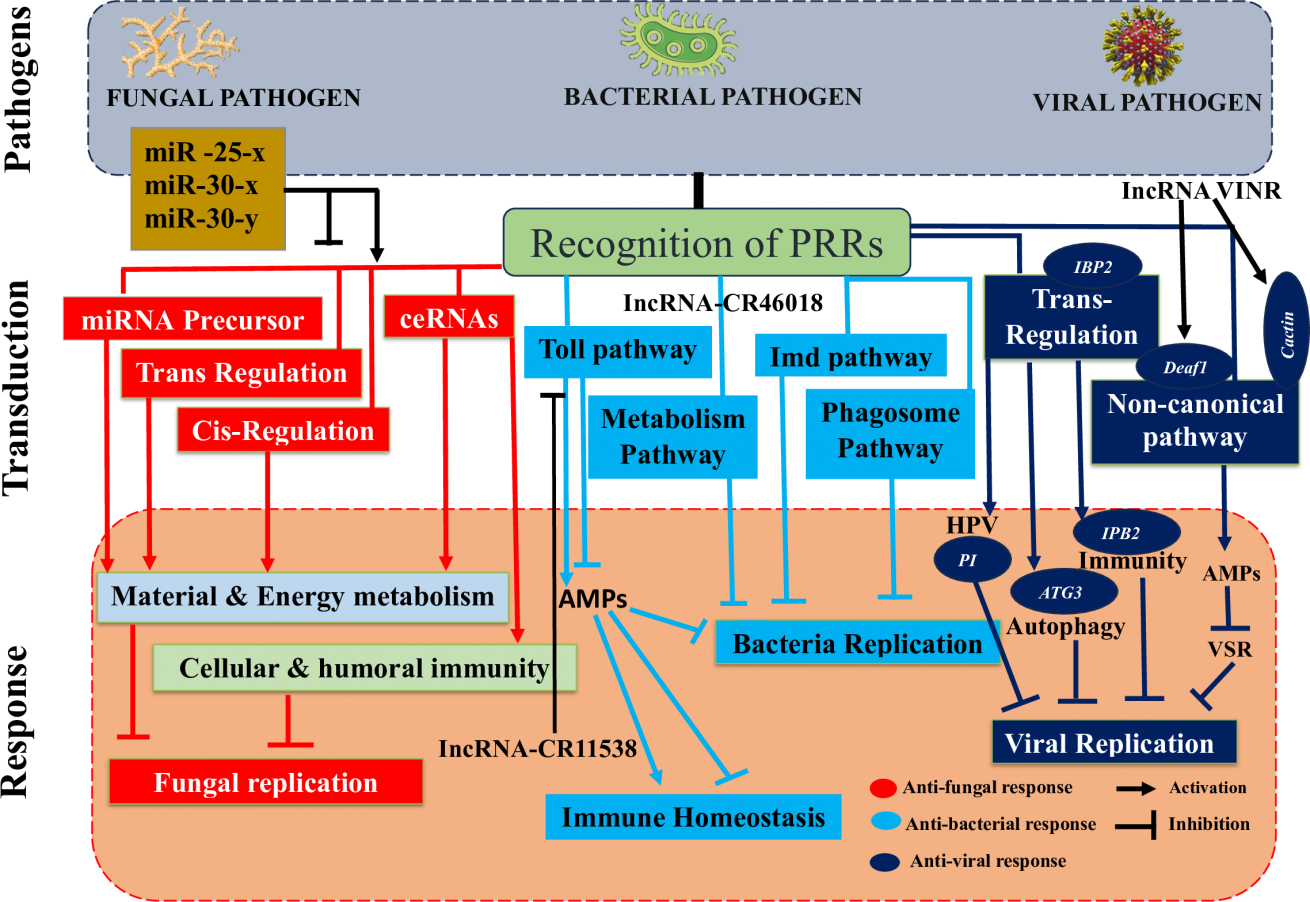
Insect immune defense is altered by lncRNAs in response to microbial invasions. Many insect lncRNAs, which control nearby genes in cis and trans, interact with miRNAs, or function as miRNA precursors, were altered as a result of the fungal invasion of insects. The majority of those trans- and cis-acting molecules stimulate cellular and humoral immunity as well as mechanisms for the metabolism of materials and energy, aiding in the management of the infection. By the enhancement of the Toll pathway, phagosome pathway, or metabolic process, lncRNAs prevent bacterial replication. Insect lncRNAs also mask the essential Toll pathway elements, reducing the production of AMPs and preventing immunological overactivation in response to bacterial invasion. Insect lncRNAs favorably trans-regulate insect genes implicated in cellular and humoral immune-related pathways (PI, ATG3, IBP2 etc.) during the viral invasion of insects (HPV, autophagy, immunity etc.). In order to compensate for the failure of the RNAi pathway, viral suppression can also be achieved by activating a noncanonical pathway. Deployed lncRNAs either directly target the transcription factor Deaf1 and the RNA polymerase II (RNAPII) for the transcription of AMPs to control the viral replication or indirectly target the virulence suppressor of RNAi (VSR) and the ubiquitination of cactin in the nucleus.

Mostly insect lncRNA responses to fungal stress were the focus of some papers that did examine interactions between fungus and insects (**Figure 5**) (1). The western honeybee, *Apis mellifera*, has a wide range of domestic uses, including the production of honey and crop pollination. *Nosema ceranae*, a fungus that forms spores and is obligately intracellular, may infect many different kinds of insects, including honeybees (221). Infection with *N. ceranae* significantly changed the expression of lncRNAs in *A. mellifera*, showing 4,749 conserved and 1,604 new lncRNAs (1). Some differentially expressed long noncoding RNAs (lncRNAs) controlled gene expression in cis and trans or acted as miRNA precursors or competitive endogenous RNAs (ceRNAs) functioning as miRNA sponges, activating the host’s vital signaling pathways and infection control (222). One of the most important ideas was that the differentially expressed lncRNAs in *A. mellifera* may restrict *N. ceranae* by interacting with miR-25-x, miR-30-x and miR-30-y due to their sponge ability. But additional experimental research is needed since the bioinformatic prediction of this lncRNA and miRNA binding is restricted (1).

#### 6.2.2 During bacterial pathogen invasion

There is some evidence that lncRNAs can modulate insect immunological responses, namely the Toll immune response to bacterial infections (**Figure 5**) (1). Dif and Dorsal genes are crucial components of Toll immune signaling in insects (223). These two effectors can initiate the transcription of AMPs, which can then be used to eliminate infections. *Drosophila* lncRNA CR46018 expression was approximately ten-fold higher after *M. luteus* infection (1). The bulk of upregulated genes were enriched in the Toll and Imd signaling pathways, as shown by RNA-seq study of *Drosophila* infected with *M. luteus* and overexpressing the lncRNA CR46018. The Toll pathway was strengthened by the interaction of CR46018 with the transcription factors Dif and dorsal, as predicted by bioinformatics and verified by RNA-immunoprecipitation studies (1). These data point to lncRNA-CR46018 being a positive regulator of the Toll signaling pathway and a vital component for *Drosophila* survival. Furthermore, lncRNA-CR46018 can up-regulate genes involved in the phagosome pathway and down-regulate genes involved in metabolic regulation. The latter pathway appears to be an appropriate target for insect-derived lncRNAs during insect-pathogen interactions. Previous research has shown that *M. luteus* infection in *Drosophila* links immunity and metabolism by way of the *Drosophila* lncRNA CR44404 (lncRNA-IBIN) (224). Further study is needed to determine how lncRNAs of insect origin regulate this pathway to improve insect defenses. Alternately, lncRNAs may downregulate immune effectors in order to exert unfavorable control over insect immunological responses and avert potentially damaging immune activation late in an infection. In order to minimize unchecked immunological activation, a recent study found that the lncRNA-CR11538 inhibited the transcription of AMPs in the latter stage (24 h) of *Drosophila* infection with *M. luteus* by decoying Dif/dorsal away from the AMP promoter and adversely influencing the Toll signaling pathway (225).

#### 6.2.3 During viral pathogen invasion

Insects rely heavily on the RNA interference (RNAi) pathway in their immune system to fight off viral infections (**Figure 5**). The RNAi pathway, however, has been demonstrated in multiple studies to be ineffective against viral replication (226). It is obvious that by encoding RNAi suppressors, viruses can escape being hampered by insect RNAi defense mechanisms. Examples of RNAi cancellers have been found in a variety of plant and insect RNA viruses (227). However, no RNAi suppressors were previously associated with arboviruses until the recent study by Zhang et al. (228). During *Drosophila* infection with *Drosophila* C virus (DCV), the antiviral lncRNA VINR accumulated in the nucleus due to the viral RNAi suppressor’s failure to prevent the upregulation of antiviral lncRNAs (1). LncRNA VINR’s capacity to bind to cactin, preventing its degradation by the ubiquitin-proteasome and promoting noncanonical antiviral and AMP defense, reduced viral multiplication (228). Viral suppression of primary antiviral RNAi immunity in *Drosophila* may have prompted a counter-counter-defense strategy involving lncRNAs. Long noncoding RNAs regulate gene expression through cis and trans-acting mechanisms (229). A comprehensive examination of lncRNAs associated with Rice black-streaked dwarf virus (RBSDV) infection in the midgut of *Laodelphax striatellus* revealed that 176 differentially expressed lncRNAs affected all predicted and differentially expressed mRNA targets in a trans manner (230). Although KEGG pathway analysis identified significantly enriched pathways like valine, leucine and isoleucine degradation, purine metabolism, fatty acid elongation and others as the most significantly enriched pathways of those trans-regulated genes during RBSDV infection, the Human papillomavirus infection pathway (which is essential for viral infection) was significantly enriched (1). Consequently, it may have a role in the infection of *L. striatellus* midgut by RBSDV (230). The expression levels of eight lncRNAs were discovered to be altered and RT-qPCR confirmed the expression of two coexpressed targets in the KEGG-predicted Human papillomavirus pathway (230). Another important function of one of the lncRNA’s targets, a protease inhibitor (PI), is in antiviral and cancer prevention (231). It’s intriguing that both the lncRNA MSTRG15394 and the PI that it targets were strongly expressed. Knockdown of MSTRG15394 or PI substantially enhanced the expression patterns of RBSDV replication-related genes, S5-1, S6 and S9-1, suggesting that these proteins could impede the accumulation and proliferation of RBSDV in the *L. striatellus* midgut (230). As a result of *B. mori* cypovirus (BmCPV) infection, lncRNAs produced by BmCPV mostly affected trans-regulation of the expression of mRNA targets in silkworm larvae (9). Many genes involved in vital processes, including autophagy (ATG3), apoptosis (PDCD6) and immunological response (IPB2), were simultaneously targeted by these differentially expressed lncRNAs, as shown by analysis of the network of differentially expressed lncRNAs and mRNAs (1). MSTRG.20486.1, the most abundant lncRNA, may trans-target several genes, including ATG3, PDCD6, MFB1, IPB2 and VPS52 (9).

## 7 Conclusions and future perspectives

It’s obvious that insects have highly effective immune systems. Phagocytosis and parasite encapsulation are the examples of cellular responses, whereas the release of antimicrobial peptides into the hemolymph is an example of humoral response. Invading pathogens are recognized by receptors such as peptidoglycan recognition proteins (PGRPs), β-glucan recognition proteins (β GRPs) and Toll-related proteins (Trps). These receptors activate many signaling pathways, such as the Toll, Imd and JAK-STAT pathways. However, the precise mechanism used by each infection and the final outcome of each case remains mostly unclear. Particularly true with viral illnesses. It follows that there will be numerous revelations to be made by future research on insect immunity. Insect-pathogen interactions now involve a new player: ncRNAs. Although the host evolves new defense strategies, the pathogens keep coming up with new ways to sneak in. This is because of the long history of coevolution between hosts and infectious agents. Because of this, illustrating the significance of ncRNAs in insect-pathogen interaction at the immunological level is of great scientific importance. In conclusion, we provided a brief overview of the main signaling routes and auxiliary immune systems in insect defense mechanisms. We also reviewed the most up-to-date information on the function of ncRNAs, in particular microRNAs and long noncoding RNAs, in regulating the immunological response of insects to pathogen invasion.

The innate immune response is a major aspect of insect disease defense, which also relies on other systems. Lack of learned or acquired defenses is what is meant by the term “innate immunity.” However, there is some evidence that *Drosophila* which have been exposed to *Streptococcus pneumoniae* at a sublethal dose once may be able to survive a second lethal dose. Although not all microbial challenges trigger this primed response, the naturally existing fly pathogen *Beauveria bassiana* may also confer a distinct protection against the fungus upon a second encounter (18). These results point to the flexibility of insect immune systems and the possibility that insect hemocytes exhibit an activation response similar to that of human leukocytes. *Drosophila* research has contributed the majority of our understanding of insect innate immunity through genetic studies of the antimicrobial peptide response, paving way for the study of Toll-like receptors, which play an important role in the innate immune response of mammals. New host antiviral genes and receptor molecules that detect viral infection can be found with the use of genetic screening in the future. Insects and their pathogens have coevolved, thus understanding their interactions is essential. As a result, it is essential to replicate findings from studies conducted with the fruit fly *Drosophila* in other insect species (232).

## Author contributions

DM, TB, JK, IS, NM, AP, GS, PK: conceptualization, writing original draft preparation and supervision. HD, MA, PPS, PM, UM, PS, VS: preparation of figures, and table. DM, TB, JK, IS, SH, SN, YY: conceptualization, preparation of figures, supervision, review and editing. All authors read and approved the manuscript.
