# Supplementary material for: Insect-pathogen crosstalk and the cellular-molecular mechanisms of insect immunity: uncovering the underlying signaling pathways and immune regulatory function of non-coding RNAs
Source: Front Immunol. 2023 Aug 24;14:1169152. doi: 10.3389/fimmu.2023.1169152 (PMC10491481; doi:10.3389/fimmu.2023.1169152)
Supplement: Supplementary file 1 [file DataSheet_1.pdf]

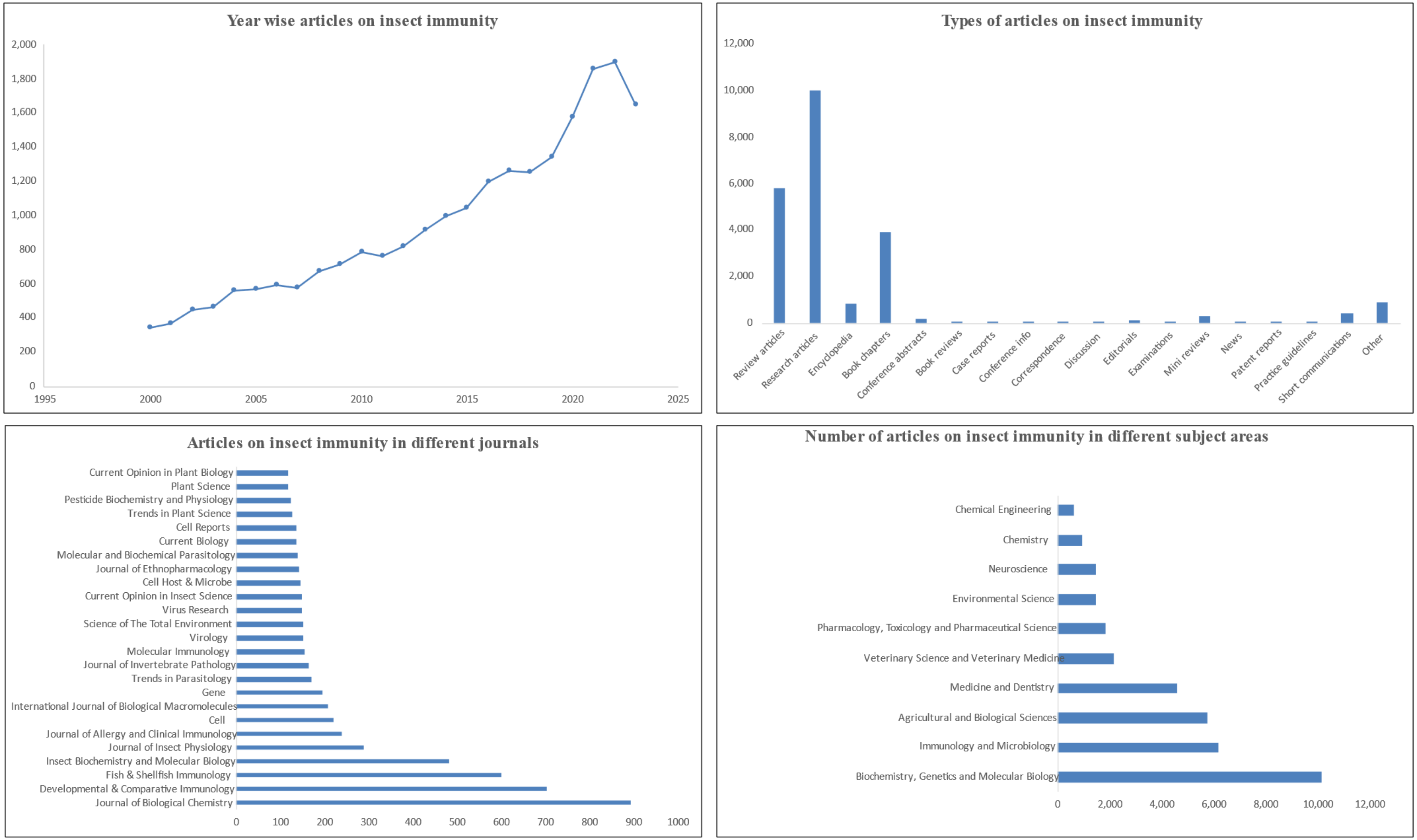

Dominance of insect immunity studies and signaling pathways over the years. This figure illustrates the growing prominence of studies focused on insect immunity and related signaling pathways over time.
